# The Role of Technical and Vocational Education relative to Academic Schooling in the Transition of the Youth to Labour Markets in Kenya

**DOI:** 10.12688/gatesopenres.16354.1

**Published:** 2025-07-28

**Authors:** Bonface Mujuri, Immaculate Kathomi

**Affiliations:** 1 Zetech University, Nairobi, Nairobi County, 00200, Kenya; 2 Egerton University, Njoro, Nakuru County, 20115, Kenya

**Keywords:** Kenya, Human Capital, Academic Education, Employment, Youth, Technical and Vocational Education& Training, Labour markets.

## Abstract

This paper explores the role of Technical and Vocational Education & Training (TVET) relative to academic education in the transition of youths to the labor market in Kenya. Kenya’s education system has experienced tremendous changes and diversification over the years, from replacing the old curriculum with the 8-4-4 system, and later transitioning to a competency-based curriculum, not to mention an overhaul of TVET via the Technical and Vocational Education and Training Act No. 29 of 2013. Due to these reforms, enrolment in TVET institutions has progressed upward over the past five years. The growth of the TVET sector has been promoted to help curb the rising rates of youth unemployment, but the evidence of the effectiveness of reforms to date has been sparse. The study provides evidence on two issues: (i) the effect of TVET on youth employment relative to academic education and (ii) the structure and status of the TVET sector in Kenya. We used quantitative and qualitative methodologies to generate the evidence. Findings demonstrate that youth with a TVET background have strong prospects of securing employment than those without TVET skills. However, the data from KIIs reveal gaps in the TVET sector that hinder graduates from competing effectively with their university counterparts in the labor market. The study recommends a review of the curriculum to promote holistic TVET training and match it with current technological innovations.

## 1. Introduction

Education plays a critical role in human capital development and contributes to the country’s increased earnings. Supporters of the human capital theory argue that a relationship exists between education and human capital accumulation ( Faggian et al., 2019). For this reason, Kenya has prioritized school enrollment at all levels, including free and compulsory primary and secondary education. Forming a proper alignment with the Education for all initiative, a central element of the Millennium Development Goals ( UNESCO, 2005). Education and training constitute an investment in human capital that is expected to yield future returns in income and earnings for the individual and society promoting economic growth ( Abraham & Mallatt, 2022). Human capital entails knowledge, skills, competencies ( OECD, 2001), and individual’ health in a nation ( Brian, 2007). Education and training are the primary channels of investing in human capital. The International Labor Organization (ILO) report on global employment trends (2020) shows that the participation rate of young people aged 15-24 years in the labor force has declined. The report also states that a fifth of young people aged 15-24 are not in employment, education, or training. This means this proportion of youths are not contributing to developing their economies. Globally much attention has been given to TVET sector. A report on strategy for Technical and Vocational Education and Training (TVET) by UNESCO (2015) attributes TVET as a potential area that solved the rising rates of youth unemployment globally. However, ILO (2020) states that vocational training tends to change much faster than the problem-solving skills taught in universities. This has implications on the employability of the TVET trainees. Moreover, vocational training is more likely to expose youths to employment in jobs that are at risk of automation. Manpower Group (2018) survey shows that in 43 countries, skilled traders in technical fields like electricians, welders, and mechanics were among the most challenging jobs to fill. This is reflected in enrolment rates to TVETs globally; for instance, Sub-Saharan Africa accounts for 1 percent of enrolments in TVETs. Therefore, the modernization of TVETs is crucial, especially in developing countries, to keep up with the changing digitalization of economies.

Among the seventeen Sustainable Development Goals (SDGs), Education is the Fourth Sustainable Development Goal (SDG). The aim is “Ensuring inclusive and equitable quality education and promoting lifelong learning opportunities for all” ( UN General Assembly, 2015). Subsections 4.3 and 4.4 specifically mention access to quality technical and vocational education being important for the employability of the youth. Section 4.3 states, “Equal access to affordable technical, vocational and higher education,” while section 4.4 states, “Increase the number of youth and adults who have relevant skills, including technical and vocational skills, for employment, decent jobs, and entrepreneurship” (United Nations Educational, scientific and cultural Organization, UNESCO, 2018). Developed countries like Australia have 24% of its population aged 16-64 and 46% of ages 15-19 years actively engaged in vocational education, showing that a large percentage of its population is accessing the vocational education system. Australia is one of the countries that have a well-developed vocational education system and strong linkages with employers. In the Kenya context, it would be important to understand the level of linkages by evaluating the level of employability of trainees from TVET institutions.

Kenya has the highest unemployment rate in East Africa at 39% ( KAM, 2021) ^1^. The Kenyan Government, faced with a growing population combined with unemployment and unmet skills demands, has chosen to expand the country’s education and training system. By 2018, the number of public educational institutions had increased to 94,399, a 4.5 percent increase from 2017 ( TVETA, 2020). The number of accredited bodies providing TVET grew by 16.7 percent to 2,289 between 2017 and 2018 ^2^.

In Kenya, the TVET sector has seen tremendous reforms in recent years. Enrolment increased at all educational levels, except for universities, with formal TVET accounting for the most significant increase of 32.3 percent between 2017 and 2018. Sessional Paper no. 14 (2012) ^3^ aimed to increase enrolment in TVET by 20 percent by 2023. The Government has intentionally committed resources to the TVET sector to revitalize, improve access, and increase enrollment rates in the TVET institutions. To mitigate this challenge, the Government seeks to create 1.3 million jobs by 2022 through the Manufacturing Pillar of the Big 4 Agenda. The government of Kenya views a strong technical and vocational education and training (TVET) system as an enabler of the Big 4 Agenda, Vision 2030, and the realization of sustainable development goals. A 2015 report by the Organization for Economic Co-operation and Development (OECD) on labor market mismatch and labor productivity indicates that many companies are having trouble in acquiring the right talent.

The majority of vocational training in the informal *Jua Kali* sector is provided by traditional apprenticeships, especially in the production and service sectors. It is estimated that 40 percent of young people in Kenya acquire vocational skills through this form of training ( MOEST, 2012). First, the study aims to examine the effect of TVET on youth employment relative to academic education. The second objective is to investigate the status of the TVET sector in Kenya. The study is timely as the Government is implementing the Competency based Curriculum (CBC) at the lower level of education both at the primary and secondary level. Therefore, strengthening TVET institutions will create synergy and ensure a smooth transition from primary and secondary education to TVET programs. This study distinguishes itself from the rest because it focuses on the skills acquired and their demand in the job market. Previous studies on TVET in Kenya focused on enrollment rates and the transition from secondary school to TVET institutions. This study demonstrated that it is not only the number of enrollments in the TVET institutions that matters but also the ability of the TVET graduates to apply the skills acquired in the job market. The graduates can only apply the skills after graduation; they can secure formal or informal employment. This study interviewed key informants from the key institutions regulating TVETs and responsible for developing policies and those tasked with developing TVETs curriculum. Other key informants included the principles of major TVET institutions in the eight regions formally known as provinces in Kenya.

## 2. TVETs Ecosystem and Employment numbers in Kenya

TVET institutions have played a critical role in equipping the youth with skills that have enabled them to get employment. As noted by the TVETA authority, in some situations, former polytechnics or other non-university institutions had been merged solely through an administrative change of Statutes. The emergence of so many upgraded polytechnics or former TVET schools converted into more university-like institutions had caused concern with some lacking specialized intermediate technical professionals to link to industrial requirements resulting in a shortage of skills in some fields. This has led to the increased unemployment rate of graduates ^4^. However, in the recent years, reforms in the education sector have been initiated by the Kenyan government through the Ministry of Education. One of these reforms includes intentional commitment of resources by the government to TVET institutions to promote the access and increase enrollment to these institutions. These reforms being implemented specifically by the Technical and Vocational Education and Training Authority (TVETA) which was established under the TVET Act No.29 0f 2013 to perform duties of regulating and coordinating training in the TVETs in Kenya. The act also highlights guiding principles that ensure there is access, equity, quality and relevance in training. The principles also insist that training is to be offered to all Kenyans without discrimination on grounds such as religion, disability, social or economic background. The Act also states that all institutions and trainers in the institutions shall be accredited, licensed and registered for quality regulations ( TVET ACT, 2013) ^5^.

### 2.1 Employment numbers in Kenya, 2006-2021

**Figure 1.**
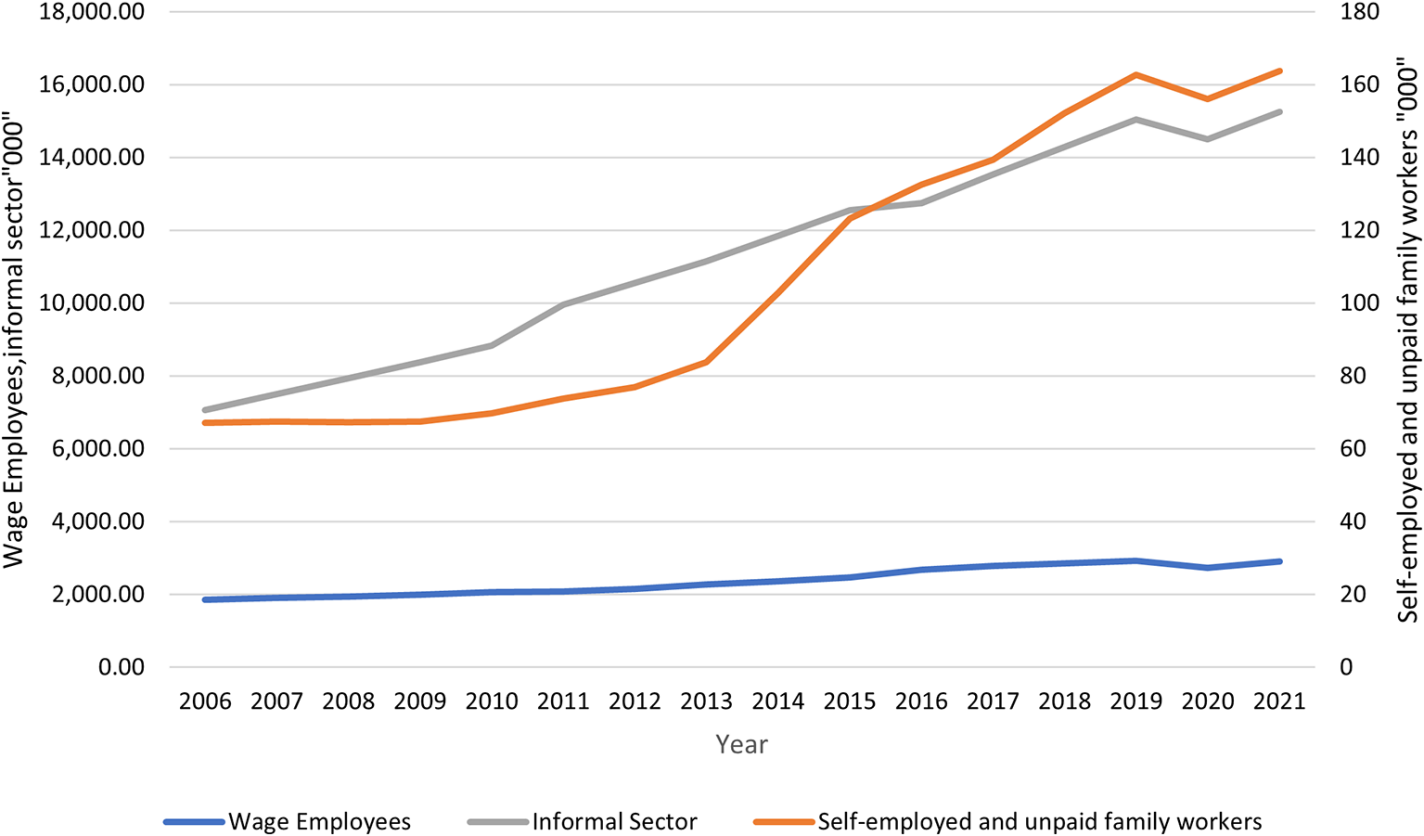
Total employment, 2006 – 2021. Source: Own construction, KNBS Economic Surveys 2021.

Figure 1 shows that wage employment, informal and self-employment have been increasing over time since 2006. Wage employment increased from 1857.6 in 2006 to 2928.4 in 2019 before falling to 2741.1 in 2020 and later rising to 2907.3 The decline in wage employment in 2020 can be attributed to the outbreak of COVID-19, which disrupted economic activities, hence cutting down employment in many sectors. Comparing the three sectors, informal creates the most employment compared to wage and self-employment. This could be due to the fact that the majority of the people do not have the skills required to be absorbed in wage employment.

## 3. Literature review

### 3.1 Theoretical review

Bohlander et al. (2001) defines human capital as the knowledge, skills, and capabilities of individuals that have economic value to an organization. On the other hand, the Organisation for Economic Cooperation and Development ( OECD, 2001) describes human capital as the knowledge, skills, competencies, and attributes embodied in individuals that facilitate the creation of personal, social and economic well-being. Tallarigo (2000) also defines human capital as capabilities, knowledge, skills, and experience, all of them embodied in and inseparable from the individual. The study focused on human capital as the knowledge, skills, competencies, experience, and attributes individuals have that contribute to translation and absorption in the job market. Thus, human capital development is any activity that increases the quality of the employee. Training is a primary mechanism by which human capital is developed. Marimuthu et al. (2009) describes it as the knowledge and training required and undergone by an employee that increases the individual’s capabilities in performing activities of economic values.

The theory of human capital was formalized by Schultz (1961) and developed further by Becker (1964). Human capital theory advocates that education or training imparts useful knowledge and skills to workers which in turn increase their productivity and incomes ( White, 2017). Becker distinguishes between specific human capital and general human capital. Specific human capital includes expertise acquired through education and training which is specific to a particular firm, firm-specific or context-specific skills. On the other hand, general human capital (general skills) is knowledge gained through education and training that is valuable across the board, such as reading and writing. Becker views human capital as similar to physical means of production, such as factories and machines.

One can invest in human capital through education, training, and medical treatment, and one’s outputs depend partly on the rate of return on the human capital one owns. Thus, human capital is a means of production into which additional investment yields additional output. The more the knowledge and skills one acquires, the more are the chances of getting engaged in the production in form of employment. A number of authors have criticized the human capital theory for being too simplistic in its analysis of employee productivity and have argued that education alone cannot lead to organizational productivity but must be complemented by other variables. Levin and Kelley (1994) pointed out that economists and other social scientists have overestimated the payoffs from increased education and ignored complimentary inputs such as training which must exist for education to improve productivity. According to Thurow (1975), productivity is mainly characteristic of jobs rather than of workers; employers use education credentials to select workers because better-educated workers can be trained for specific jobs more quickly and at a lower cost than their less-educated peers. Spence (1973) also argues that education may simply be a market signal of the potential productivity of a worker since there is hardly any other way for firms to determine the productive attributes of a worker. Notwithstanding these criticisms, “Becker’s human capital theory has been resilient and still remains the principal theoretical construct that is used for understanding human capital investment, both from the perspective of the individual and the firm” (Bassi & McMurrer, 2006).

### 3.2 Empirical review

Several studies about TVET education in Kenya have been done. However, these studies have mostly concentrated on the enrolment in the TVET institutions. The studies ignored the aspect of skills acquired by these graduates. The researchers also did not explore the number of graduates who have transited to the job market and whether the skills acquired are demanded in the market.

Kiplangat (2020) found that the cost of vocational education was high and therefore contributed to low enrolment of students in Elgeyo-Marakwet county, Kenya ^6^.

Wasike and Maiyo (2020) concluded that the type and number of courses offered by a TVET institution determine student enrollment levels. The study also concluded that budget adequacy in TVET institutions had a significant relationship with the overall enrolment of students in the TVET institutions. ^7^ All these studies focused on enrolment without considering the quality of the graduates and whether the skills acquired in these institutions are needed in the job market.

Wanjala and Malechwanzi (2016) found that technical and other educational institutions need to improve the quality of education if they are to be significant players in the world’s economic arena. Lack of the quality and necessary skills for the job market had previously resulted to the TVETs graduates failing to secure employment.

Shaorshadze and Krishnan (2013) conducted a study on Technical and Vocational Education and Training in Ethiopia. The study outlined the outcomes of the TVET graduates relative to the counterfactual of no having gone through the TVET and how to carry out the impact evaluation of such.

A study by Nafukho (1998) established that the Kenyan government’s efforts to promote vocational training in the educational system as a way of addressing the unemployment problem had not been very successful. He called this a vocational school fallacy. Graduates from IT equipped with technical and vocational skills could not find employment in either formal or informal sectors This has since changed in Kenya. This is contradicted by the World Bank (2018) findings that indicated technical and vocational training enhances the critical chances of employability among the youth. In the United States, the distribution of incomes within schooling groups has been rising ( Levy & Murnane, 1992).

Nickell (2004) using the IALS data, considers how differences in the distribution of incomes across countries are affected by the distribution of skills and by institutional factors including unionization and minimum wages. While union coverage is statistically significant, he concludes that “the bulk of the variation in earnings dispersion is generated by skill dispersion”. This underscores the impact of skills on earning which can come because of one getting employed. This could be one factor that employers could be looking for before they employ a TVET graduate. If one does not have the required skills, will not secure an employment and therefore will have low income.

Mankiw et al. (1992) in their study on Contribution to the Empirics of Economic Growth established that education increases the human capital inherent in the labour force, which increases labour productivity and thus transitional growth towards a higher equilibrium level of output. The argument is that the more one educated is the more the likely the person will get employed.

Hanushek and Woessmann (2008) investigated the role of cognitive skills in promoting economic well-being, with a particular focus on the role of school quality and quantity. They concluded that there is strong evidence that the cognitive skills of the population rather than mere school attainment are powerfully related to individual earnings, to the distribution of income, and to economic growth. This shows that shows that education and more particularly the skills attained contribute greatly to one getting employed.

Nason (2019) conducted a study “Youth Unemployment among Graduates of Tertiary Institutions in Kenya the Case of Africa Inland Church Jericho”. The study established that one of the causes of youth unemployment is lack of employability skills among the youth as shown by the unemployment rate of only 12.5% of those who had TVET education. However, for those with other kinds of education, their level of unemployment was high with 93.75% of them being diploma holders and 80% of degree holders were not employed.

Awiti et al. (2019) conducted a survey of employers and employees in the formal and informal sectors to determine entry-level skills among youths aged 18-30 years in employment in Kenya established that there is a skills mismatch whereby the gap between the skills by the youth entering the workforce and the job market had widened due to the growing dominance of the services industry. The study was conducted generally regarding the youth and did not look at the TVET graduate’s aspect.

Munene and Wanjiku (2020) conducted a study on Contribution of Fisheries to Job Creation Among the Youth in Kenya. They found that skill mismatch was one of the contributors of the employment among the youth. Employment rates was however high among the TVET graduates compared to the University counter parts. This study however did not use the disaggregated data and did not demonstrate that the employment of TVET graduates was as a result of possessing the necessary job-needed skills.

### 3.3 Conceptual framework

**Figure 2.**
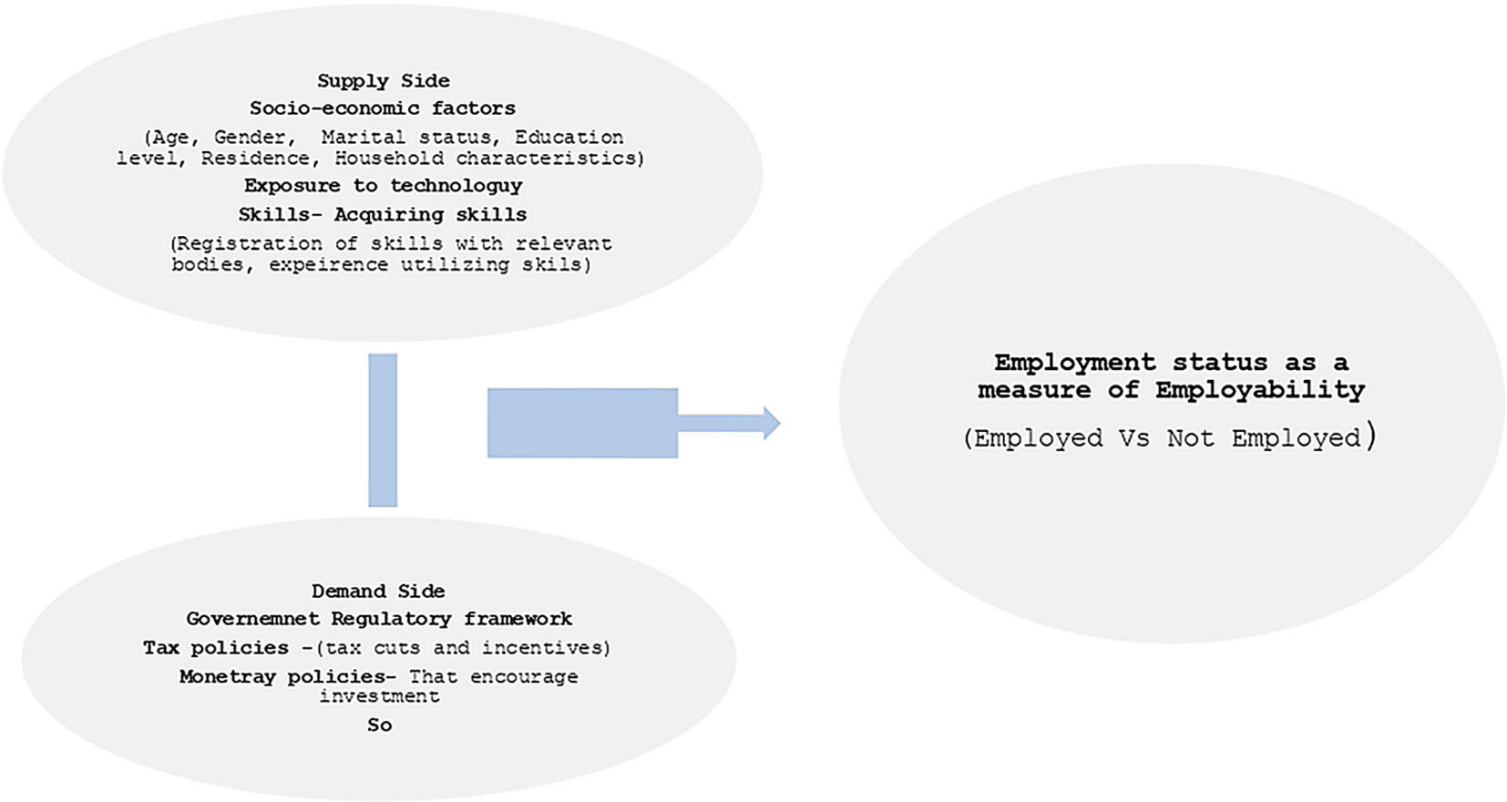
Conceptual framework. Source: Author’s own construction.

Employability is defined by the combination of skills, personal attributes, and knowledge that enable an individual to obtain, maintain, and transition between different forms of employment ( Husain et al., 2020). According to Williams et al. (2015), the definition of employability keeps on evolving over time. Artess et al. (2017) argues that an individual’s employability does not guarantee that they are employed, an individual maybe employable but unemployed. However, the sole reason for being employable is to seek employment. Therefore, employment status of an individual can be used as an indicator for employability where the success will be securing employment ( Artess et al., 2017). However, this cannot be used as the only determinant of employability. Employability should refer to the possession of skills that are in demand in the job market. So, it is used as a regressor in an employment equation. Possession of a TVET certificate is an indicator of employment. In addition, employability keeps on progressing and diminishing depending on an individual and the sector they are involved in over time ( Yorke, 2006). Holmes (2013) bases the definition alongside the employer where he defines employability as the set of skills and experience that employer look for in individuals. Skills, attributes knowledge attainment and characteristics of an individual form an integral part of gauging the employability of an individual ( Teijeiro et al., 2013). On the other hand, employer’s needs, industrial needs and government policies are variables that influence the employability of a set of individuals of a country ( Clarke, 2018). Arguably, employability involves interaction of variables from an individual perspective to external factors. In macroeconomics the demand side of labour market provides for the employers needs while the supply side shows what employees feed the labour market ( Arntz et al., 2019). Figure 2 below shows the conceptualization of this study from a macroeconomics perspective. The study adopts employment status as the indicator for measure of the employability of an individual where being employed is treated as the success. In order to be employable, there exist an interaction of the supply and demand factors of the labour market. In the supply side, the employee’s attributes skills and characteristics affect employability while the demand side employers need affect the employability. The quantitative study meant to measure the extent of employability of TVET graduates where there is control of factors on the supply side. Specifically, the characteristics of socio-economic and technology exposure were adopted in the quantitative study. The qualitative study explored the factors on the supply side in order to understand the state of employability of TVET graduates in Kenya ( Figure 2).

## 4. Empirical strategy

### 4.1 Data sources

The study used the Kenya Integrated Household Budget Survey (KIHBS) data collected by the Kenya National Bureau of Statistics (KNBS) for the year 2005/2006. The choice of KIHBS 2005/2006 over later versions, such as KIHBS 2015/2016, was intentional and driven by the specific research objective: to investigate the influence of Technical and Vocational Education and Training (TVET) on youth transitions into the labour market. KIHBS 2005/2006 contained detailed components on education pathways, including disaggregated data on technical and vocational training, which were not comprehensively captured in the 2015/2016 edition. Therefore, it provided a more appropriate and targeted dataset for answering our research questions related to TVET. The survey covered 13,430 households stratified by district and urban-rural classification and included extensive modules on education, employment, household characteristics, and other socioeconomic indicators allowing for a multi-dimensional understanding of the variables influencing youth employment outcomes. The reason for including urban and rural classifications is to provide a more nuanced understanding of the findings. This distinction helps capture contextual differences in socioeconomic conditions, service accessibility, and labor market opportunities, thereby adding depth and credibility to the analysis. The data also shows coverage of all the 47 counties in Kenya. In addition, data was collected from Key Informant Interviews (KIIs) for the qualitative study to enrich the quantitative analysis with contextual insights and to triangulate findings from the econometric model for deeper policy relevance. To ensure regional representation, a stratified sampling approach was adopted. A total of 16 Technical and Vocational Education and Training (TVET) institutions were selected, with two institutions randomly sampled from each of the eight former provinces in Kenya. In recognition of the institutional diversity within the TVET sector, the sample was further stratified by institutional hierarchy. Each of the four identified hierarchies within the TVET system was represented by four institutions, ensuring balanced representation across different levels. Prior to the main data collection, a pre-test of the research instruments was conducted to assess their validity and reliability. Feedback from the pre-test informed necessary revisions, enhancing the clarity and effectiveness of the tools used in the Key Informant Interviews (KIIs). KIIs provided first hand perspectives from institutional actors across the TVET landscape, helping to interpret observed statistical relationships, highlight implementation challenges, and uncover institutional factors influencing youth transitions that are not captured in household-level data. *STATA* software was used for econometric analysis while *EXCEL* was used to organize data for descriptive and thematic analysis. Patterns emerging from Key Informant Interviews (KIIs) are presented in narrative form.

### 4.2 Econometric model

The study used linear probability model (LPM) ( Huang, 2022) to estimate effects of TVET education transitions of youth from schools to labour market. A binary logistic model was used to check the robustness of the results obtained. In the study the outcome variable is the employment status of the youths which takes a value of one when a youth is employed and a value of zero otherwise. We consider a youth employed by wage as the success category otherwise the youth is unemployed indicating failure to transit to the labour market. The model is specified as follows ( Huang, 2022).

$$\;p\left((Y=1|X=x)\right)=\beta_{0}+X^{1}\beta +e$$ (1)

From this general equation we built two sets of models; the first model estimates the probability of a youth getting waged employment given that the youth goes through the academic education system, controlling for socio-demographics. The second model estimates the probability of a youth getting wage employment given that the youth goes through the technical and vocational training track, again controlling for socio-demographics. The two models can be specified as;

$$p\left(Y_{\text{youth secures wage employment}}|\text{going through academic education track},x1;X\right)=\beta_{0}+\beta_{1}x_{1}+X^{1}\beta +e$$ (2)

$$p\left(Y_{\text{youth secures wage employment}}|\text{goes through tvet education track},x2;X\right)=\beta_{0}+\beta_{2}x_{2}+X^{1}\beta +e$$ (3)

Where X is the vector for other control variables, β is the vector of coefficients corresponding to each control variable, $\beta_{1}$ is the coefficient for the education level variable through academic education and $\beta_{2}$ is the coefficient for the education through TVET education and e is the error term. To address potential shortcomings of the LPM, such as heteroscedasticity and predicted probabilities outside the 0-1 range, we conducted a robustness check using a Binary Logistic Regression model. This allowed us to confirm the direction and significance of the results obtained from the LPM while adhering to the theoretical assumptions of logistic models for binary outcomes.

### 4.3 Description of variables

Table 1 explains how the variables were used and coded when carrying out the analysis in the study. From the table the dependent variable (employment status of the youth) was operationalized as a binary (dummy) variable where 1 indicates a youth in waged employment and 0 otherwise. Waged employment was chosen as the key indicator of successful labor market integration, consistent with approaches by various authors like ( Coletti & Pasini, 2023; Zwysen, 2019) and the nature of data used in the study. It provides a clear, quantifiable outcome and reflects the formal engagement of youth in the workforce. The primary independent variable is technical and vocational education, measured as a categorical variable distinguishing between youths with no vocational training and those who attended government colleges, commercial colleges, or village vocational centers. This variable captures not only whether the youth participated in vocational training but also the type of institution, which has been shown to influence employment outcomes due to differences in curriculum quality, industry linkages, and skill relevance ( Ndile, 2018). To provide a comparative perspective, the study also includes the highest level of academic education, coded as a categorical variable with nine levels ranging from no formal education to university degree. This variable allows for an examination of how formal academic qualifications compare with vocational training in influencing employment. The inclusion of academic education as a predictor is supported by studies showing that while academic pathways are often valued in formal employment sectors, they may not always provide the practical skills required in the labor market ( Bol et al., 2019). Several socio-demographic variables are included as control variables, based on their known influence on employment outcomes, which included; age, gender, marital status, residence, religion, school management and literacy. Age is categorized into three groups (15–17, 18–21, and 22–25 years), reflecting key stages in youth development and transition to work ( Wood et al., 2018). Place of residence coded as 1 for rural and 0 for urban, captures the geographic dimension of employment access. According to Mabiso and Benfica (2019) youth in rural areas may face more significant challenges in accessing training institutions and job opportunities.

**Table 1.** Definition of variables.

Variable name	Definition
Employment status of the Youth	Dummy Variable showing status of youth in waged employment where 1= waged employment of youth is the success, 0 otherwise
Highest Level of education (Academic education)	Categorical variable with nine categories of Education levels: 0 = No formal education, 2 = Kenya Certificate of primary Education/Certificate of Primary Education, 3 = Kenya Certificate of Education/Kenya Certificate of Secondary Education, 4 = Kenya Junior Secondary Examination, 5 = Kenya Advanced Certificate of Education/East African Certificate of Education, 6 = Certificate, 7 = Diploma in private institution, 8 = Diploma, 9 = Degree
Technical and Vocational Education	Categorical variable with four categories; 0 = No vocational education, 1 = government college, 2= Commercial college, 3 = village vocational centres
Age	Categorical variable with three categories; 1 = youths of ages 15-17 years, 2 = ages 18-21 years, 3 ages 22-25 years
Gender	Dummy variable where 1 = Male, 0 = female
Place of residence	Dummy variable where 1 = youths living in rural areas, 0 = urban areas
Marital status	Dummy variable where 1 = youths who are married, 0 = single
Religion	Dummy variable where 1 = Christians who are protestants, 0 = otherwise
School management	Categorical variable where; 1 = School managed by government, 2 = managed by a religious body, 3 = managed by private organization, 4 = managed by community
Whether a youth is able to read	Dummy Variable where 1 = yes, that is when a youth can read, 0 = no a youth who cannot read

## 5. Results and Discussion

This section presents descriptive results and regression results. Table 2 shows the descriptive results of all the variables in the model.

**Table 2.** Summary statistics.

Variable name	Number. of observations (N)	Mean	Standard deviation	Minimum value	Maximum value
*Employment status of the Youth*					
Unemployed	16041	0.8876	0.3159	0	1
Employed	16041	0.1124	0.3159	0	1
*Highest Level of education (Academic)*					
No formal Education	14100	0.4646	0.4988	0	1
Kenya Certificate of Primary Education	14100	0.3619	0.4806	0	1
Kenya Certificate of Secondary Education	14100	0.1325	0.3390	0	1
KJSE		0.0026	0.5116	0	1
KACE/EAACE	14100	0.0014	0.0376	0	1
Certificate	14100	0.2525	0.1569	0	1
Diploma Private Institution	14100	0.0040	0.0635	0	1
Diploma	14100	0.0042	0.0646	0	1
Degree	14100	0.0035	0.0588	0	1
*Technical and Vocational Education*					
No Technical and Vocation Education	14090	0.9085	0.2883	0	1
Government College	14090	0.0289	0.1675	0	1
Commercial College	14090	0.0449	0.2071	0	1
Village Vocational centres	14090	0.0176	0.1318	0	1
*Age*					
15-17 years	16041	0.3225	0.4674	0	1
18-21 years	16041	0.3862	0.4869	0	1
22-25 years	16041	0.2913	0.4544	0	1
*Gender*					
Female	16041	0.5053	0.4998	0	1
Male	16041	0.4947	0.4998	0	1
*Place of residence*					
Urban	16041	0.3124	0.4635	0	1
Rural	16041	0.6876	0.4635	0	1
*Marital status*					
Single	16041	0.8384	0.3681	0	1
Married	16041	0.1616	0.3681	0	1
Religion					
Other Religious Affiliations	16041	0.5384	0.4985	0	1
Christian	16041	0.4616	0.4985	0	1
*School Management*					
School managed by Government	16041	0.3632	0.4809	0	1
School managed by religious body	16041	0.0162	0.1263	0	1
School managed by private organization	16041	0.0350	0.1839	0	1
School managed by community	16041	0.5856	0.4926	0	1
*Whether a youth is able to read*					
No	16041	0.1039	0.3051	0	1
Yes	16041	0.8961	0.3051	0	1

### 5.1 Descriptive analysis

Table 2 shows the number of observations, the mean, standard deviation, minimum value and maximum values for each variable in the study while further disaggregating each variable to the respective categories. From the table on average 88.76 percent of the youths are unemployed while 11.24 percent of the youths are in wage employment. According to the United Nations, Human development Index (2017), Kenya had unemployment rate at 39 percent making it the highest among its neighboring East African countries ( Farah & Ali, 2018). The situation is especially common among the youths, where 85 percent of the unemployed people are youths ( KNBS, 2018).

Notably, on average most of the youth aged 15-25 years reported not having formal education. The percentage decreases as the level of education goes high. In lower levels of Kenya certificate of primary education reported an average of 36.19 percent of youth. However, youth with certificates were 25.25 percent, which is more than youth who had Kenya certificate of Secondary Education. Interestingly there are more youth who have attained diplomas at 0.4 percent compared to youth with degrees at 0.3 percent.

The results in Table 4 also reveal that most of the youth have not gone through technical and vocational education at 90.85 percent. Out of the youth who have gone through the Technical and vocational education, the highest number of youth have gone to the commercial colleges at 4.49 percent, followed by government colleges at 2.89 percent. The least number of youth have gone to the village vocational centres at 1.76 percent. The youth considered in this analysis were youth ranging from 15 years of age to 25 years of age. Out of these youth 32.25 percent had 15-17 years, 38.62 percent had 18-21 years while 29.13 percent had 22-25 years. Female youths were 50.53 percent while male youth were 49.47 percent.

Most of the youth sampled lived in rural areas at 68.76 percent. In addition, most were single at 83.84 percent and 46.16 percent were Christians. The descriptive analysis further looked at the school management where majority of the schools revealed to be managed by communities at 58.56 percent while government managed schools accounted for 36.32 percent of the schools. Finally, majority of the youth could be able to read at 89.61 percent.

### 5.2 Regression analysis

Table 3 shows the regression results for the LPM. The coefficients and standard errors (in parentheses) are presented. Two columns are presented in the table to show the results of the two models in equations 2 and 3, where in one model the probability of a youth securing wage employment is estimated given that the youth go through the academic education track while column two shows estimate for the second model where a youth goes through the vocational schooling system.

**Table 3.** Linear Probability Model (LPM) Results: Effects of Academic Education and Technical and Vocational Education Systems on Youth Employment.

Variables	Regular Education	Vocational Education
	Coefficients (Standard Errors)	Coefficients (Standard Errors)
Highest Level of education/schooling *(Base category = No formal Education)*		
Kenya Certificate of Primary Education	-0.0168 *** (0.0058)	
Kenya Certificate of Secondary Education	-0.0619*** (0.0085)	
KJSE	0.0220 (0.0486)	
KACE/EAACE	-0.0187 (0.0660)	
Certificate	0.0838 *** (0.0165)	
Diploma Private Institution	0.1021 ** (0.0394)	
Diploma	0.1228 *** (0.0389)	
Degree	0.0459 (0.0426)	
Technical and Vocational Education *(Base category = No Technical and Vocation Education)*		
Government College		0.0735*** (0.0149)
Commercial College		0.0647*** (0.0123)
Village Vocational centers		0.0252 (0.0191)
Age *(Base category = 15-17 years)*		
18-21 years	0.0048 (0.0066)	-0.0067 (0.0064)
22-25 years	0.0614*** (0.0082)	0.0460*** (0.0079)
Gender *(Base category = female)*		
Male	0.0696*** (0.0051)	0.0704*** (0.0051)
Place of residence *(Base category = Urban)*		
Rural	-0.0899*** (0.0056)	-0.0816*** (0.0055)
Marital status *(Base category = single)*		
Married	-0.0966*** (0.0079)	-0.0900*** (0.0079)
Religion *(Base category = Other religious affiliations)*		
Christian	0.0198 *** (0.0049)	0.0180*** (0.0049)
School Management *(Base category = School managed by government)*		
School managed by religious body	-0.0025 (0.0190)	-0.0115 (0.0188)
School managed by private organization	-0.0164 (0.0133)	-0.0274*** (0.0133)
School managed by community	0.2071*** (0.0065)	0.2040*** (0.0066)
Whether a youth is able to read *(Base category = No)*		
Yes	0.0009 (0.0162)	-0.0157 (0.0165)
Number of observations	*14,100*	*14,090*
*F*-Statistics ( *p*-values)	*143.97 (0.000)*	*192.33 (0.000)*
R- squared	*0.1554*	*0.1508*
Root MSE	*0.2944*	*0.2951*
Significance levels: ^*^	p<0.010, ^**^ p<0.050, ^*^p<0.100.	

From Table 3 the total number of observations under the regular (academic) education was 14100, while the total number of observations for vocational education was 14090. The R squared value for the model on regular education was 0.1554, indicating that the 15.54 percent of the variations in the model are accounted for by the variables in the model. Similarly, 15.08 percent of the variations in the model where a youth goes through vocational education are accounted for by the variables in the model.

The categories for the highest level of education attained that had coefficients that were statistically significant include: Kenya certificate of primary education, Kenya Certificate of Secondary Education, Certificate levels, and diplomas while the base category was ‘ *no formal education’.* We find that when a youth attains the Kenya Certificate of Primary Education only, the certificate lowers their chances of getting wage employment by 1.68 percent relative to persons with zero academic education. The Kenya Secondary Education Certificate also lowers chances of getting wage employment by 6.19 percent compared to youths with no education. This can be attributed to the fact that it is expected for the youths to proceed to higher levels of education and obtain skills that can help them get wage employment. In line with Morsy and Mukasa (2019), undereducated youths majorly feature in informal sectors of employment whereby in most cases the terms of employment are casually rated. Interestingly, when compared to youths with no formal education youths who obtain certificates at any level increase their relative chances of getting waged employment by 8.38 percentage points, while those who obtain diplomas from private institutions increase the relative chances of getting wage employment by 10.21 percentage points. Youths who obtain diplomas from non-government institutions increase their chances of getting wage employment by 12.28 percent compared to youths with no education, showing that they are better off in securing wage employment compared to youths who attend the private institutions for diplomas. Although the coefficient for the degree level is not significant the youths who obtain degrees have a 4.59 percent higher chance of securing wage employment which is much lower compared to youths who obtain certificates and diplomas. Universities have been associated with preparing graduates for white-collar jobs which are growing scarce especially in low and middle-income countries ( Ponge, 2013). The number of students graduating from universities exceed the available jobs in the economy, therefore lowering the chances of securing employment ( Gudo et al., 2011).

From the table it is clear that youth who obtain technical and vocational education have higher chance of securing wage employment compared to youths who do not obtain any technical and vocational education. Youth who attend the government technical or vocational colleges have 7.35 percent higher chance of securing wage employment. The results are consistence with the findings by Shaorshadze and Krishnan (2013). Youth who attend the commercial (private) colleges have 6.47 percent higher chance of getting wage employment relative to their counterparts without technical education background. Government colleges are better endowed in terms of available like laboratory equipment making youths graduating from these institutions very well knowledgeable compared to private institutions ( Ngware et al., 2022). Although not statistically significant, the coefficients on vocational education at the village level, have 2.52 percent higher probability of securing wage employment compared to those who do not have any technical or vocational training.

Youth aged 22-25 years and have gone through regular/academic education system have 6.14 percent chance of securing wage employment compared to those with age 15-17 years. Youth aged 22-25 years and having technical and vocational education have 4.60 percent higher chance of getting wage employment than those aged 15-17 years. At lower ages, they are still building on their skills through training and education ( Breen, 2005).

The results further reveal that male youth have a better chance of securing wage employment than female youths in both systems of education. Male youth who attend regular education have of 6.96 percent higher chance of securing wage employment compared to female. In technical and vocational education male youth have a 7.04 percent higher chance of securing wage employment compared to female youths. These findings are in line with findings by Baah-Boateng (2016). This is also reflected in the enrolment numbers that show gender disparity in favour of male. However, cultural roles and marital status of women are drivers that hinder such youths from securing employment ( Kamau & Wamuthenya, 2021).

In both cases of the education system, living in rural areas lowers the chances of youth securing wage employment. Youth living in rural areas have regular education have 8.99 percent lower chances of getting wage employment compared to those living in urban areas. Youth living in rural areas and attend technical and vocational education have 8.16 percent lower chances of securing wage employment compared to youth living in urban areas. According to Elder et al. (2015), youth in rural areas are more likely to engage in casual wage jobs that are seasonal and inconsistent hence the lower chances of being in employment compared to those in urban settings.

Table 4 shows the regression results of the LPM model where two columns are presented; one column represents the model for the female youths’ sub-sample, while the other represents the male sub-sample. From Table 4 the number of observations for the female sub-sample is 6978 while the number of observations for the male sub-sample is 7112. The R squared for the female sub-sample was 0.1296 showing that 12.96 percent of the variations in the model for the female sub-sample are explained by the variations of the variables in the model. However, R squared for the male sub-sample was 0.1894 showing that 18.94 percent of the variations in the model are explained by the variations in the variables in the model for the male sub-sample.

**Table 4.** LPM Results: Effects of Technical and Vocational Education on Employment by Gender.

Variables	Female sub-sample	Male sub-sample
	Coefficients (Standard Errors)	Coefficients (Standard Errors)
Technical and Vocational Education ( *Base category = No Technical and Vocation Education)*		
Government College	0.0346 (0.019)	0.1009*** (0.0226)
Commercial College	0.0692*** (0.0145)	0.0539*** (0.0204)
Village Vocational centers	0.0229 (0.0224)	0.0237 (0.0313)
*Age (Base category = 15-17 years)*		
18-21 years	-0.0044 (0.0084)	0.0002 (0.0096)
22-25 years	0.0368*** (0.0101)	0.0435*** (0.0121)
*Gender (Base category = female)*		
Male	Omitted	Omitted
*Place of residence (Base category = Urban)*		
Rural	-0.0845*** (0.0068)	-0.0791*** (0.0084)
*Marital status (Base category = single)*		
Married	-0.1446*** (0.0086)	0.1005*** (0.0157)
*Religion (Base category = Other Religious Affiliation)*		
Christian	0.0234*** (0.0063)	0.0158* (0.0076)
*School Management (Base category = School managed by government)*		
School managed by religious body	-0.0180 (0.0228)	-0.0228 (0.0297)
School managed by private organization	-0.0284 (0.0171)	-0.0296 (0.0198)
School managed by community	0.1663*** (0.0087)	0.2367*** (0.0096)
*Whether a youth is able to read (Base category = No)*		
Yes	0.0281 (0.0213)	-0.0579 (0.018)
*Number of observations*	*6978*	*7112138.24*
F-Statistics ( *p*-values)	*86.42(0.000)*	*138.24 (0.000)[*
R Squared	*0.1296*	*0.1894*
Root MSE	*0.2612*	*0.3179*
Significance levels: ^*^	p<0.010, ^**^ p<0.050, ^*^p<0.100.	

Clearly, for all the control variables the chances of securing wage employment favour male youth compared to female. Female youth who attended commercial colleges have a 6.92 percent higher chance of securing employment compared to those without technical and vocational education. Male youth who attended government colleges have a 10.09 percent higher chance of getting wage employment.

Female youth who are aged 22-25 years have a higher chance of wage employment which 3.68 percent higher than that for age group 15-17.

Male youth aged 22-25 years have 4.35 percent higher chances of securing wage employment than that for age group 15-17. Living in rural areas lowers the chances of securing wage employment for both genders however, the situation is worse for female youth where the probability of getting wage employment is 8.45 percent lower compared to that of male of 7.91 percent.

Interestingly the study finds out that marriage decreases the chances of getting wage employment by 14.46 percent relative singles for female youth. The chances of securing wage employment for married male youth is 10.05 percent higher than that for single males.

Marginal effects were computed from the probit estimates presented in (Annex 8).

For technical and vocation education, the base level is no technical and vocational education and the marginal effect of government college is 0.0611 showing that the expected probability of wage employment is higher for the youth with technical and vocation education from government college than those with no technical and vocational education by 0.0611 when the other independent variables are held constant at their means. Expected probability of wage employment is also higher for the youth with technical and vocational education from commercial college and Village Vocational centres is higher than those with no technical and vocational education by 0.0401 and 0.0149 respectively when the other independent variables are held constant. Expected probability of wage employment for the youths aged 22-25 years is higher than those aged 15-17 years by 0.0367 holding other independent variables constant. Same case is observed with youths aged 18-21 years who have higher probability of 0.0042 of getting wage employment than those aged 15-17. Finally, the expected probability of wage employment for the male youth is higher than that of female youths by 0.0741 holding all other independent variables constant at mean.

Probit model was also run where the county dummies were included capturing the 47 counties in Kenya and results presented in table 6 (Annex 9). The results shows that when county-specific variables are taken into account, the lpm results change but not much.

### 5.3 Findings from key informant interviews

Information from KIIs was obtained through structured questionnaires. Each category of key people involved in the TVET space were interviewed. The institutions were sampled from the eight former regions namely; Nairobi, central, Eastern, North Eastern, Rift Valley, Coast, Western and Nyanza. In each region at least two institutions were sampled. The institutions sampled cover all levels of TVET colleges that is National polytechnics, technical training institutes and vocational centres.

KIIs carried out in the institutions sought to establish the opportunities that are there for TVET students during their time in the college to prepare them for job market. Further, we sought views from key informants in the institution on the readiness, challenges and solutions that can help them make better interventions to promote easy transition for their graduates to the job market. Two national polytechnics were sampled, 7 technical training institutes and 7 technical and vocational centres were sampled. National polytechnics in Kenya are high in ranking of TVETs. Technical and vocational centres are the last in ranking of TVETs in the country.

**5.3.1 Thematic findings**

i) *Graduating students are more numerous in high ranking TVET institutions than in low ranking TVET Institutions*

The KII revealed that there is a variation in the number of students graduating from TVET colleges depending on the level of ranking of a TVET college. In National Polytechnics (NPs) the frequency of graduation is much higher than in TVCs and TTIs. In NPs, graduations are held at least once every year with an average of over 500 students graduating while in TTIs and TVCs graduations are held when the institution establishes there is enough students on average about 200 to graduate. Some TTIs and TVCs take about two years before holding a graduation ceremony. For instance, in Meru National Polytechnic an average of 700 students graduates per year while in Uhuru TVC the average graduating number of students is 300. This variation is brought about by the difference in enrolments rates where more students get enrolled in NPs compared to TTIs and TVCs. The results are consistent with the findings by Kiplagat et al. (2018) where he established that completion rates among students in NPs and TTIs is higher compared to TVCs due to institutional factors, where management in NBPs is better controlled than in *TVCs*.

ii) *In most institution students are responsible for securing industrial attachment on their own*

Industrial attachments expose students applying what they have learnt in real life and also teaches them the job. It is important for students to get industrial attachment before graduating to expose them to the details of a typical working environment. In all the KIIs, most institutions do provide introduction letters to help students seek attachment, however they have the sole responsibility to find places where they can be attached. However, few institutions revealed that they have a liaison attachment officer, who is responsible for creating networks with industries and referring students to apply to such industries so that they can get attachment opportunities.

Institutions are making efforts to collaborate with CSOs, companies and businesses to expose students to opportunities: Some institution shave created partnerships with companies, reputable Jua kali centres and non-Governmental organizations to help students get attachment, and even employment. For instance, Waondo VTC collaborates with local reputable shops in tailoring and metal works to attach students to them so that they can gain better hands-on skills through apprenticeship even after going through training in school. This however, is not very common among many institutions.

iii) *Issue of soft skills is a challenge among TVET graduates*

TVET graduates lack the soft skills to help them navigate through job searching making them less likely to get jobs especially where they are competing with the university graduates for the same roles. This issue has also been demonstrated in many studies like: Ochieng and Ngware (2021), Ondieki et al. (2019) among others. Policy recommendations on how to integrate whole youth development in order to fully equip TEVT students and make them equally competitive in the labor market have been made.

iv) *Changing technology does not match the efforts to change curriculum*

The digital era is taking course in many countries. Many respondents revealed that TVET graduates are at a risk of irrelevance with digital economies coming up if the government does not regularly review the curriculum to include the topics of evolving technology. For instance, one of the respondents stated that books being used to teach some technical sources were written 20 years ago may sometimes have irrelevant information from what is in the market.

v) *Unfavourable terms of entrepreneurship or self-employment makes it difficult for most graduates to establish their businesses despite having the right skills*

This study also found out from KII that even though self-employment is the greatest thing can be used by TVET graduates to solve the unemployment issue among the youth, the procedures one must go through before starting up a business in the country are sometimes unfavourable and may scare away youths from setting up their own businesses. Even though the government has set up interventions like *Uwezo* fund, the conditions for giving these loans are not reasonable in that they can only be accessed by groups which may not in turn perform well compared to giving such loans to individuals.

vi) *Collapsing industries in the country that mostly absorb TVET graduates due to unfavourable business operating environment*

Most industries are shutting down and moving to countries with better environments to run their businesses this poses a major challenge to TVET graduates since they are the ones absorbed by these industries mostly especially for their production departments.

## 6. Conclusions and Recommendations

The study established that youth who obtain technical and vocational education have high chances of securing wage employment compared to those who do not obtain any technical and vocational education. Age is another factor that influences wage employment with the youth aged 22-25 years having a high chance of getting wage employment compared to those aged 15-17 years. Gender also plays a critical role as male youths have high probability of getting wage employment compared to female youth. Other factors that were found to influence youth wage employment includes: area of residence, marital status, religion and school management. Other contributing factors based on KIIs include: low completion rate due to delayed graduation of students, lack of coordinated internship and industrial attachments. This denies graduates from putting skills learnt in class into practice. In addition, lack of internship and industrial attachment leaves graduates with no room for them to gain experience which is a requirement for one to be employed. Other factors affecting transition to employment are lack of soft skills, change in technology while curriculum remain unchanged to match the new technologies, rigid policies that are unfavourable to the young graduates who would want to start self-employment by starting own business and lack of enough employment opportunities due to low industrialization in the country. The political class promoting the growth of TVET is a good thing for the graduates of TVET. This means that for instance lawmakers who are members of parliament are able to table and support bills that can empower the TVET graduates to get more jobs. Key informants reported that in most cases where they have collaborated with companies and businesses to offer internship, they end up retaining the intern as a permanent employee because by the end of the internship they are usually fit to occupy positions permanently especially for technical work like plant operators.

Based on these findings several policy implications can be adopted. First, there is a need for the government to develop policies that emphasize the enrollment of youth in TVET institutions, as this increases their chances of securing wage employment compared to those with no formal education. Second, there is need for adoption and emphasize of affirmative action that ensures both male and female youth get equal chances of getting employed. Apparently male youth have higher chance of getting wage employment compared to male youth. Subsequently, there is need to support and equip government schools in terms of resources and infrastructure to accommodate more youths as results indicate that that youth who attend government colleges have higher chance of getting wage employment than those from commercial, Village Training centres and those without Technical and Vocational education. In addition, there is need for intentional provision of internship and apprenticeship programs for the TVET graduates. Government and other employers should offer internship and apprenticeship opportunities to the TVET graduates in technical places such as agricultural and manufacturing. This will expose more TVET graduates to the job market and therefore, give them equal opportunity for competitiveness for jobs. Lastly, there is need for the Curriculum update to match current job market changes: The ministry of education in collaboration with TVETA need to develop a curriculum that matches the current market needs and that is aligned with the current changes in the technology. This will help graduates to acquire the relevant market skill.

## Data Availability

The data (Kenya Integrated Household Budget Survey 2005-2006) used in this study is available at Kenya National Data Archive (KeNADA) under and is open access. One just needs to create account and will be able to access the data.

## Footnotes

^1^

https://kam.co.ke/why-tvet-is-central-to-economic-development/

^2^

https://www.nationalskillsgateway.go.ke/training_programs.html?applicableId=1&pageTitleId=3&pageId=7&applicableHeader=4

^3^

http://repository.kippra.or.ke/bitstream/handle/123456789/490/Sessional%20paper%20no%2014%20of%202012.pdf?sequence=1&isAllowed=y

^4^

https://www.tveta.go.ke/2021/08/31/why-kenya-must-operationalise-technical-universities-as-pathways-for-tvet-graduates/

^5^

https://www.kttc.ac.ke/images/access_to_information/Technical_and_Vocational_Education_and_Training_Act_No29of2013_.pdf

^6^

https://ir-library.ku.ac.ke/handle/123456789/21948

^7^

http://erepository.kibu.ac.ke/bitstream/handle/123456789/2539/Maiyo_Utility%20of%20government%20initiatives%20in%20technical%2C%20vocational%20and%20training%20institutions%20on%20student%20enrolment%20in%20Bungoma%20County%2C%20Kenya.pdf?sequence=1&isAllowed=y
